# Risk Assessment and Mapping of Hand, Foot, and Mouth Disease at the County Level in Mainland China Using Spatiotemporal Zero-Inflated Bayesian Hierarchical Models

**DOI:** 10.3390/ijerph15071476

**Published:** 2018-07-12

**Authors:** Chao Song, Yaqian He, Yanchen Bo, Jinfeng Wang, Zhoupeng Ren, Huibin Yang

**Affiliations:** 1 State Key Laboratory of Remote Sensing Science, Institute of Remote Sensing Science and Engineering, Faculty of Geographical Science, Beijing Normal University, Beijing 100875, China; songc345@163.com (C.S.); yanghb@mail.bnu.edu.cn (H.Y.); 2 School of Geoscience and Technology, Southwest Petroleum University, Sichuan 610500, China; 3 Department of Geology and Geography, West Virginia University, Morgantown, WV 26505, USA; heyaqian1987@gmail.com; 4 State Key Laboratory of Resources and Environmental Information System (LREIS), Institute of Geographic Sciences and Natural Resources Research, Chinese Academy of Sciences, Beijing 100101, China; wangjf@lreis.ac.cn (J.W.); renzp@Lreis.ac.cn (Z.R.)

**Keywords:** HFMD, spatiotemporal zero-inflated modeling, climate and socioeconomic factors, spatiotemporal mapping, Bayesian Hierarchical method

## Abstract

Hand, foot, and mouth disease (HFMD) is a worldwide infectious disease, prominent in China. China’s HFMD data are sparse with a large number of observed zeros across locations and over time. However, no previous studies have considered such a zero-inflated problem on HFMD’s spatiotemporal risk analysis and mapping, not to mention for the entire Mainland China at county level. Monthly county-level HFMD cases data combined with related climate and socioeconomic variables were collected. We developed four models, including spatiotemporal Poisson, negative binomial, zero-inflated Poisson (ZIP), and zero-inflated negative binomial (ZINB) models under the Bayesian hierarchical modeling framework to explore disease spatiotemporal patterns. The results showed that the spatiotemporal ZINB model performed best. Both climate and socioeconomic variables were identified as significant risk factors for increasing HFMD incidence. The relative risk (*RR*) of HFMD at the local scale showed nonlinear temporal trends and was considerably spatially clustered in Mainland China. The first complete county-level spatiotemporal relative risk maps of HFMD were generated by this study. The new findings provide great potential for national county-level HFMD prevention and control, and the improved spatiotemporal zero-inflated model offers new insights for epidemic data with the zero-inflated problem in environmental epidemiology and public health.

## 1. Introduction

Hand, foot, and mouth disease (HFMD), mainly occurring in young children, is a worldwide infectious disease caused by enterovirus and can lead to death [1]. The most obvious symptom of HFMD is that patients have small herpes or ulcers in positions of hand, foot, and mouth on the body. HFMD is mainly transmitted through air and close contact [1,2,3]. In China, HFMD is a leading infectious disease and has been formally incorporated into the national monitoring system, since May 2008 [2]. From 2008 to 2013, China’s HFMD incidence rate increased remarkably from 37.6/100,000 persons to 139.6/100,000 persons [3]. HFMD has posed a serious threat to China’s public health security. However, the spatiotemporal epidemics of HFMD across Mainland China are still unclear.

Previous studies have revealed that HFMD is strongly associated with climate environmental factors including temperature [4,5], humidity [6,7], precipitation [8,9], wind speed [10,11], air pressure [12], and sunshine [13]. Climate conditions not only impact the reproduction and transmission of the viruses causing HFMD, but also change the physical activities of children [14], which together promote the opportunity for viral contact among young children [15]. Socioeconomic factors may also modify the climate effects on HFMD [16,17,18]. However, few studies concerned both socioeconomic and climate factors for HFMD risk assessment and mapping, especially on the spatiotemporal scales. In addition, previous HFMD studies in China mainly focused on small regions, such as Guangdong [19], Shandong [20], Beijing [21], Shenzhen [10], and Sichuan [22]. Whereas, with regard to Mainland China [16,17,23,24,25], no studies have established spatiotemporal final scale risk maps of HFMD at the county level, not to mention accounting for both climatic and socioeconomic factors.

Moreover, epidemiological data for disease mapping with excessive zeros is defined as a zero-inflated (ZI) problem [26], because most diseases are on rare conditions. China’s HFMD surveillance data suffered a serious ZI problem because they were collected at the county level, the smallest national administrative division unit [16]. Ignoring the ZI problem in epidemiological data could drop important disease characteristics, reduce disease relative risk (*RR*) mapping accuracy and increase uncertainty [26]. Former studies have considered ZI effects for other diseases [27,28,29,30], however, to our best knowledge, no studies have considered the ZI problem for HFMD risk assessment and mapping.

Zero-inflated models have been widely used for handling count data with excessive zeros [31,32], among which zero-inflated Poisson (ZIP) and zero-inflated negative binomial (ZINB) are the two most popular ones [33]. In addition, spatiotemporal models have gained popularity for disease risk assessment and mapping in epidemiology [34,35]. Arab et al., have presented a review of an advanced methodology combining spatiotemporal and zero-inflated models for spatial and spatiotemporal epidemiological data with excessive zeros [26]. Recently, the spatiotemporal ZI models have drawn much attention in environmental epidemiology and public health. For instance, Musenge et al. utilized Bayesian spatiotemporal zero-inflated models for HIV/TB in South Africa [29]. Amek et al. applied a zero-inflated binomial model for spatiotemporal modeling of sparse geostatistical malaria sporozoite rate data in Kenya [28]; Musio et al. used Bayesian semi-parametric ZIP models with space-time interactions for lymphoid leukemia incidence data in France [36]. Spatiotemporal models are preferably specified within the Bayesian hierarchical modeling (BHM) framework because BHM can account for ZI effect and similarities based on the neighborhoods among space and time flexibly [34,35] and has been widely used in environmental epidemiology [37,38]. However, such spatiotemporal zero-inflated models have not been utilized to solve the ZI problem for HFMD.

To address the aforementioned shortcomings in current HFMD studies, we built spatiotemporal ZIP and ZINB models under the BHM framework, accounting for both climatic and socioeconomic covariates, using the monthly county-level HFMD cases data across the whole Mainland China in 2009. The objective of this paper is four-fold: (1) to test the effectiveness of ZI influence between spatiotemporal ZI models and traditional models, (2) to identify environmental risk factors for HFMD considering both climatic and socioeconomic aspects, (3) to fit the spatial clusters and nonlinear temporal trend of HFMD relative risks, and (4) to estimate the complete spatiotemporal risk maps at county-level in Mainland China.

## 2. Materials and Methods

### 2.1. Data and Study Area

For the study area of Mainland China, we acquired county-level monthly data including HFMD cases, climate, and socioeconomic variables for the year 2009. A total of 2310 counties were valid for analysis.

**Figure 1:**
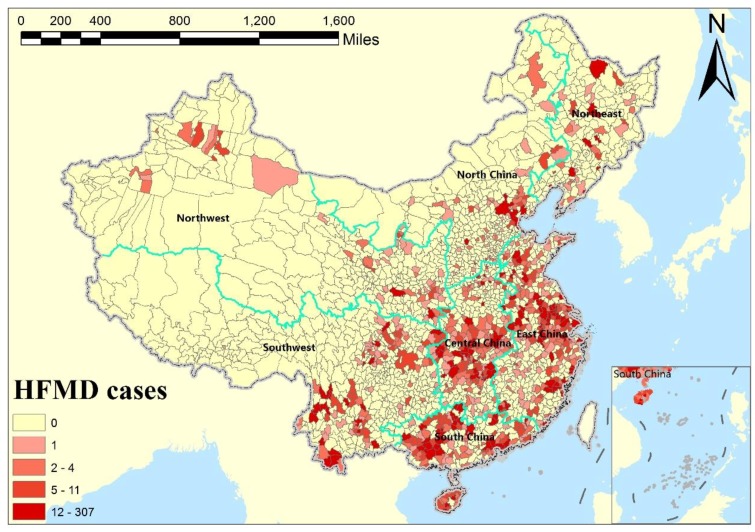
Geographical distribution of reported hand, foot, and mouth disease (HFMD) cases in Mainland China in January 2009.

HFMD case data in children aged between 0–9 years was from the China Information System for Disease Control and Prevention (CISDCP). In the year 2009, there were about 1,166,000 HFMD cases and the HFMD incidence rate was 75.84/100,000 children across Mainland China. The highest incidence rate occurred in April with 13.77/100,000 children. Figure 1 shows the geographical distribution of reported HFMD cases in Mainland China in January 2009, where a large number of areas are with “zero” occurrences. China’s HFMD epidemic data suffers a serious zero-inflation problem, thus it is necessary to consider the ZI effect in disease risk assessment and mapping.

The monthly climate data in this study was based on the raw data collected from 727 climate stations throughout China from the China Climate Data Sharing Service System [16]. Data of yearly socioeconomic variables were from the China County Statistical Yearbook, China Statistical Yearbook for Regional Economy, and China City Statistical Yearbook [39]. We included a total of six climatic variables and fourteen socioeconomic variables as the potential environmental risk factors for HFMD in this study (Supplementary File S1, Table S1).

### 2.2. Statistical Models

#### 2.2.1. Spatiotemporal Epidemic Models

Within the study area, we denote the county-level areal units as *i* = 1,..., *I* (*I* = 2310) and the months as *t* = 1,..., *T* (*T* = 12). In epidemiology, conditional to the relative risk $\lambda_{it}$, the rare disease cases $Y_{it}$ are usually assumed to be Poisson-distributed. The likelihood function in spatiotemporal Poisson model is expressed as follows [40]:

(1) $$Y_{it}\sim Poisson(E_{it}\lambda_{it})\;$$

where $E_{it}$ is the expected value for area *i* and time *t*. $\lambda_{it}$ is the target estimated variable and is explained as the standard morbidity ratio (SMR) [36]. Disease cases are usually rare or zero in areas with small populations, which leads to extreme incidence values for direct disease mapping. The SMR map can smooth the extreme outliers and give more intuitive information, and thus has been widely used for disease risk mapping [41].

With Poisson data assumption, the spatiotemporal model we applied in this study is decomposed additively into components regarding climate and socioeconomic covariates, space, and time:

(2) $$\eta_{it}=\log(\lambda_{it})=\beta_{0}+\sum_{k}^{m}\beta_{k}C_{k}+\sum_{j}^{n}\alpha_{j}SE_{j}+\mu_{i}+\nu_{i}+\gamma_{t}+\varphi_{t}\;$$

where $\eta_{it}$ is the structured additive linear predictor; $\lambda_{it}$ is estimated SMR of HFMD in space *i* and time *t*; *C_k_* is the *k*-th climatic environmental variables; *SE_j_* is the *j*-th socioeconomic environmental variables; $\beta_{0}$ quantifies the intercept fixed effect; $\beta_{k}$ quantify climate fixed effects; $\alpha_{j}$ quantify socioeconomic fixed effects; and $\mu_{i},\nu_{i},\gamma_{t},\phi_{t}$ represent main spatial and temporal random effects [34,35].

Relative risk (*RR*) is widely used to measure the risk of disease exposure to a determinant in epidemiology [42]. Risk indicator *RR* can be obtained directly using $RR=e^{\beta_{}}$. In epidemiology, an *RR* value higher than one indicates that the exposure variable is a positively correlated risk factor, lower than one means a negatively correlated risk factor, and equal to one means an unrelated factor.

Regarding the spatial components including two spatial random effects: one assumes an independent Gaussian exchangeable prior to model unstructured heterogeneity, which is $\nu_{i}\sim N(0,\delta_{\nu }^{2})$, and the other one assumes an intrinsic conditional autoregressive (CAR) prior for the spatially structured variability [43], which is as follows:

(3) $$\mu_{i}|\mu_{j\neq i}\sim N(\frac{1}{m_{i}}\sum_{i\sim j}^{}\mu_{i},\frac{\sigma^{2}}{m_{i}})\;$$

where *i* ~ *j* indicates that areas *i* and *j* are neighbors, *m_i_* is the number of areas that share boundaries with the *i*-th area, and $\sigma^{2}$ is the variance component. Spatial dependence in $\mu_{i}$ assumes the CAR prior that extends the well-known Besag model [43] with a Gaussian distribution and implies that each $\mu_{i}$ is conditional on the neighbor $\mu_{j}$ with variance dependent on the number of neighboring counties *m_i_* of county *i*.

The CAR prior model assumes that the disease incidence risk in a spatial area is derived from nearby geographical neighbors. That is, the closer the space distances, the more similar disease incidence risk is in these spatial areas. This structured spatial character is called spatial autocorrelation [44]. On the contrary, the Gaussian prior model for the unstructured spatial effect represents the spatial heterogeneity, in which the spatial areas are independent of each other.

Regarding the temporal components: the term $\phi_{t}$ is the unstructured time effect, which is specified using an independent mean-zero normal prior to the unknown variance $\sigma_{\phi }^{2}$; the term $\gamma_{t}$ represents the structured time effect and is modeled dynamically through a neighboring structure. Here, the random walk (RW) dynamic model is used as a prior for the structured time effect [45], whose prior density π is written as follows:

(4) $$\pi (\gamma_{t}|\sigma_{\gamma }^{2})\propto \exp(-\frac{1}{2\sigma_{\gamma }^{2}}\sum_{t=2}^{T}{\left(\gamma_{t}-\gamma_{t-1}\right)}^{2})\;$$

Similar to CAR, the RW prior model assumes that the disease incidence risk is influenced by adjacent time points (temporal correlation). The temporal variation of disease risk is assumed to be a smoothly varying curve, and when this structured temporal trend is nonlinear, the RW model is more suitable. The independent prior model for the unstructured temporal effect represents the temporal heterogeneity.

Similar to the *RR* calculation aforementioned, we could also obtain the local *RR*s for the structured spatial and temporal random effects with $RR_{i}=e^{\mu_{i}}$ and $RR_{t}=e^{\gamma_{t}}$, respectively. The interpretation of local *RR* is also similar. The higher the *RR*, the higher the risk. For instance, a spatially local *RR* greater than one indicates that the spatial unit is a high-risk area, an *RR* value less than one indicates that the spatial unit is a low-risk area, and an *RR* equal to one means that the risk of the areal unit is on an average level.

In addition, except for the aforementioned Poisson distribution, the negative binomial distribution is particular for delineating the distribution of positive integer count data. As HFMD cases are positive integer data, the negative binomial distribution is also suitable. The likelihood function in a spatiotemporal negative binomial model is written as follows:

(5) $$Y_{it}\sim nBinomial(E_{it}\lambda_{it})\;$$

#### 2.2.2. Zero-Inflated Models

A disproportionately large frequency of zeros in the aggregated epidemic data leads to a poor performance of Poisson models for relative risk. To overcome this issue, the so-called zero-inflated model is a promising method. A zero-inflated model is a mixture model with two components: one arising from a parent distribution and the other corresponds to the excessive zeros that cannot be accounted for by the distribution [32]. In this study, we introduced two commonly used zero-inflated models to further develop the spatiotemporal model [26]. One is the zero-inflated Poisson (ZIP) model [46], and the other one is the zero-inflated negative binomial (ZINB) model [33]. 

The ZIP model is described as follows [33,46]:

(6) $$P(Y_{it}=y_{it})=\{\begin{array}{c} p_{it}+(1-p_{it})f(0),y_{it}=0\\ (1-p_{it})f(y_{it}),y_{it}>0^{,} \end{array}\;$$

(7) $$f(y_{it})=\frac{\lambda_{it}{}^{y_{it}}}{y_{it}!}\exp(-\lambda_{it})\;$$

where $Y_{it}$ is a count variable and $\lambda_{it}>0$. *p* represents the probability of the existence of extra zeros. When *p* is 0, the model is a Poisson distribution.

Compared with the ZIP model, the ZINB model [32,33] is more reliable to explain the large dispersion structure of data. Similarly, we assume that $f(y_{it})$ following a negative Binomial distribution, the ZINB model is described as follows:

(8) $$P(Y_{it}=y_{it})=\{\begin{array}{c} p_{it}+(1-p_{it})f(0),y_{it}=0\\ (1-p_{it})f(y_{it}),y_{it}>0^{,} \end{array}\;$$

(9) $$f(y_{it})=\frac{\Gamma (y_{it}+\alpha )}{y_{it}!\Gamma (\alpha )}{\left(\frac{\alpha }{\alpha +\lambda_{it}}\right)}^{\alpha }{\left(\frac{\lambda_{it}}{\alpha +\lambda_{it}}\right)}^{y_{it}}\;$$

where $\alpha^{-1}$ is a dispersion parameter and *p* is the zero expansion parameter. When *p* is 0, the model is a negative Binomial distribution.

#### 2.2.3. Spatiotemporal Zero-Inflated Models

To evaluate the performance of incorporating ZI models in spatiotemporal modeling for our case, we built four models for comparison. These four models had the same components as Equation (2), including covariate information in the spatiotemporal process, but assuming different data distribution models. Specifically, data distributions in the four models are as follows:

The traditional spatiotemporal Poisson model (herein referred to as model 1) is given by Equation (1);

The spatiotemporal negative Binomial model (herein referred to as model 2) is given by Equation (5).

The spatiotemporal ZIP model (herein referred to as model 3) is given as follows:

(10) $$Y_{it}\sim ZIP(E_{it}\lambda_{it})\;$$

The spatiotemporal ZINB model (herein referred to as model 4) is given as follows:

(11) $$Y_{it}\sim ZINB(E_{it}\lambda_{it})\;$$

With the spatiotemporal ZI models, the SMR estimation can take into account ZI influence and comprehensively incorporate the climate and socioeconomic environmental covariates, spatial autocorrelation effect, and temporal nonlinear variations.

#### 2.2.4. Covariates Selection

Before modeling, one important step is to select representative variables from a variety of potential variables. Three criterion strategies were used for selecting the candidate climate and socioeconomic variables in this study. Firstly, the variance inflation factor (VIF) for each candidate variable was calculated to assess the multicollinearity [47]. The larger the VIF, the more severe the multicollinearity. Normally, the variables selection considers VIF < 10 as the screening standard. Secondly, we used the forward stepwise regression method to exclude the variables without statistical significance [16]. We set 0.05 and 0.1 as the threshold significance values. Finally, covariates were retained in the model unless their removal resulted in the increase of deviance information criterion value by 30 units or more [48].

### 2.3. Model Evaluation Methods

#### 2.3.1. Deviance Information Criterion

The deviance information criterion (*DIC*) method is a well-known model criterion for comparing Bayesian models’ fitness and complexity, defined as follows [49]:

(12) $$DIC=\bar{D}+P_{D}{}_{}\;$$

where $\bar{D}$ is the mean of model posterior deviance and *P_D_* is the effective number of parameters. A large $\bar{D}$ indicates a great error in the model. A large *P_D_* indicates a high complexity of the model. The smaller the *DIC* and the *P_D_*, the better. Models with smaller *DIC* indicate a better trade-off between complexity and fitness of the model.

#### 2.3.2. Conditional Predictive Ordinate

The conditional predictive ordinate (*CPO*) is defined as a leave-one-out cross-validated predictive density at a given observation and can be used to access predictive quality [50]. For continuous distributions, it is defined as follows:

(13) $$CPO_{it}=p(y_{it}^{*}|y_{f})\;$$

where $y_{it}^{*}$ is the predicted value and $y_{f}$ is the sample of observations *y*, which is used to fit the model and to estimate the posterior distribution of the parameters. In practice, the cross-validated logarithmic score (*LS*) computed from *CPO* is widely used to evaluate the predictive quality for Bayesian models. A smaller *LS* indicates a better prediction of a Bayesian model. *LS* is calculated as follows:

(14) $$LS=-\frac{1}{IT}\sum_{i=1,t=1}^{I,T}\log(CPO_{it})\;$$

#### 2.3.3. Watanabe-Akaike Information Criterion

The widely applicable information criterion (*WAIC*, also known as Watanabe-Akaike information criterion) can be viewed as an improvement on the *DIC* for Bayesian models [51]. Unlike *DIC*, *WAIC* is invariant to parameterization and also works for singular models. *WAIC* is interpreted as a computationally convenient approximation to cross-validation and is defined as follows [52]:

(15) $$WAIC=LPD+P_{W}\;$$

where *LPD* is the expected log pointwise predictive density and *P_W_* is the estimated effective number of parameters. The explanation of *WAIC* is similar to *DIC*.

### 2.4. Model Inference

A spatiotemporal model can be formalized within a Bayesian framework by simply extending the concept of the hierarchical structure, incorporating similarities of neighborhoods in terms of space and time. Our spatiotemporal hierarchical Bayesian models include three levels, namely, data distribution, spatiotemporal process, and parameter, with each level further containing a number of sub-levels. We employed four different likelihood models for the data distribution level, which are Poisson, Negative binomial, ZIP, and ZINB. For the spatiotemporal process level, we combined different sub-models to account for the spatial and temporal random effects, that is, CAR and RW, respectively. For the parameter level, we specified the inverse gamma distributions as priors for all unknown variance parameters in the Bayesian framework. We selected the non-informative priors for the parameters and their variance components, which allowed the observational data to have the greatest influence on posterior distributions without being greatly affected by the settings of priors [35]. The Bayesian models presented in this study were inferred and computed using the integrated nested laplace approximation (INLA) in R software [53]. A major advantage of using INLA is a relatively short computation time with accurate parameter estimates [54]. The R-INLA package can be directly downloaded from http://www.r-inla.org/. The core codes for these spatiotemporal models are summarized in Supplementary File 2 and have been published [35,45,54].

## 3. Results

### 3.1. Model Evaluation and Comparison

Table 1 showed the evaluation results of the four alternative spatiotemporal Bayesian hierarchical models. With the lowest evaluated values, the spatiotemporal ZINB model (model 4) turned out to be the best regarding model fitness (*DIC* and *WAIC*), complexity (*P_D_* and *P_W_*), and predictive ability (*LS*), compared with that of the other three models. Hence, the optimal model 4 is applied to HFMD spatiotemporal risk analysis and mapping. In addition, the models accounting for ZI influence (model 3 and 4) had better performance than those models (model 1 and 2) without accounting for ZI influence. This indicates that incorporating ZI effects in spatiotemporal modeling can improve the model performance for the Chinese HFMD case. Moreover, we found that model 3 (Negative binomial) is better than model 1 (Poisson), and model 4 (ZINB) is better than model 2 (ZIP), which further indicates that models considering negative binomial distribution are better than traditional disease models that only consider Poisson distribution. 

### 3.2. Environmental Risk Factors for HFMD

The optimal spatiotemporal model (i.e., model 4: ZINB) was first applied to identify the environmental risk factors of HFMD, with jointly considering disease spatial and temporal random effects variables, that is, $\mu_{i}$, $\nu_{i}$, $\gamma_{t}$, and $\phi_{t}$. Covariates selection results of the climate and socioeconomic variables accounting for multicollinearity, significance, and *DIC* are summarized in Supplementary File 3 (Tables S2–S4). Table 2 summarizes the statistics for posterior estimated parameters and *RR* values of the selected covariates in the model. The factors in the regression result were used to explain the relative risk of covariates for the entire study area, including both non-occurrence (zero-inflated) and occurrence counties.

We found that both climate and socioeconomic aspects had significant influences on HFMD incidence in China. Among climate variables, HFMD incidence risk increased with increasing temperature (*RR* = 2.02), relative humidity (*RR* = 1.12), sunshine hours (*RR* = 1.24), and wind speed (*RR* = 1.16). The hot and humid environment was an important environmental risk condition for the breeding of HFMD. Regarding socioeconomic variables, we found HFMD incidence risk increased with higher economic developed covariates including the enterprise number density (*RR* = 1.41), per capita fixed assets investment (*RR* = 1.44), and per capita GDP (*RR* = 1.22). The covariate proportion of children (*RR* = 1.14) representing the demographic aspect also had a positive risk effect on HFMD incidence, which indicated children population agglomeration could increase disease risk.

### 3.3. Temporal Risk Effects of HFMD

**Figure 2:**
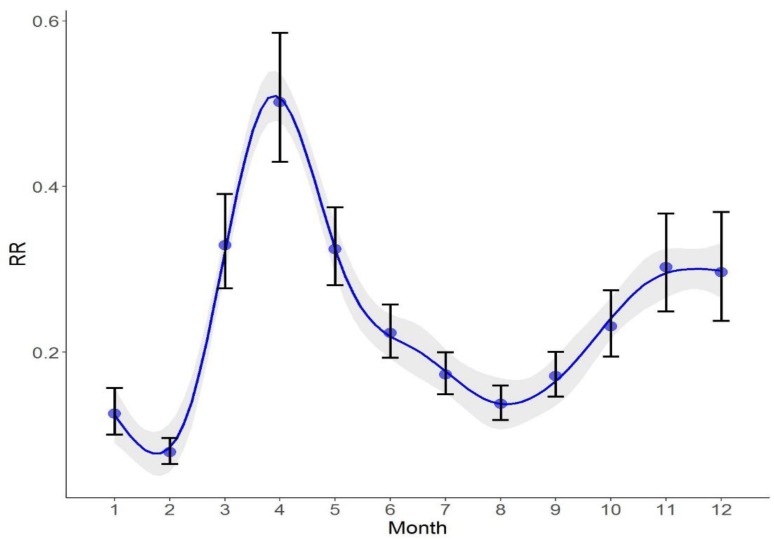
Temporal risk trend of HFMD incidence during 12 months in the year 2009.

We further used the results from the optimal spatiotemporal ZINB model (model 4) to detect the distribution of relative risk for HFMD on both spatial and temporal scales. Figure 2 illustrated the main structured temporal *RR* trend of HFMD incidence in the whole study area. We found that HFMD has obvious seasonal characteristics in Mainland China. The lowest risk occurred in February. Within 12 months, there was one peak. The highest risk occurred in April, the beginning of summer. There was also a clear increasing trend after August from fall to winter in the year 2009. 

### 3.4. Spatially Risk Effects of HFMD

**Figure 3:**
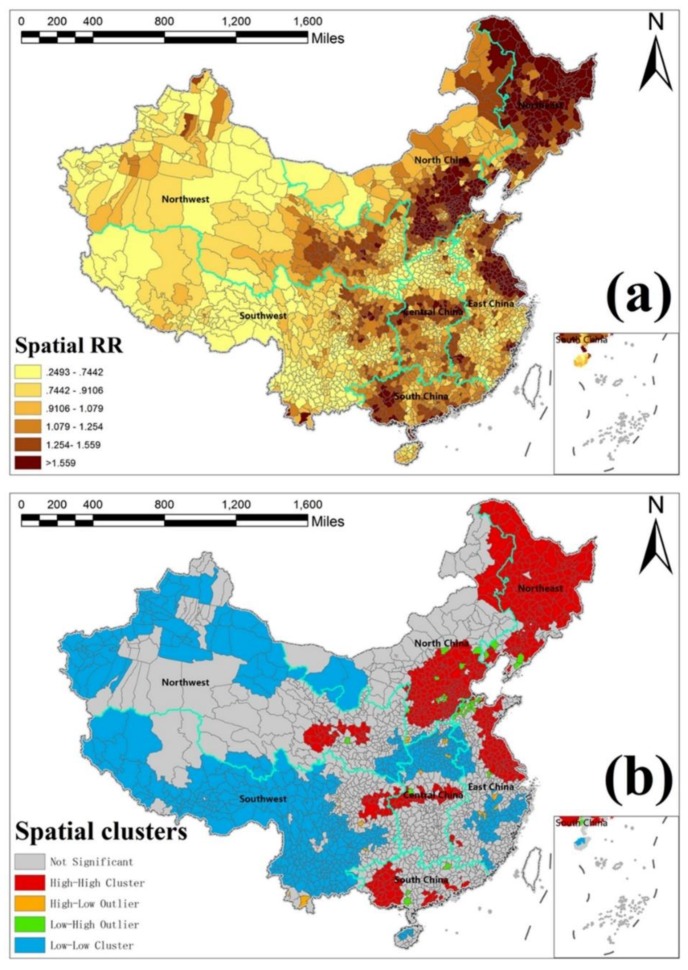
(**a**) Spatial structured relative risk (*RR*) map and (**b**) its cluster map of HFMD in Mainland China.

Figure 3a is the *RR* risk map representing the spatial structured risk distribution of HFMD incidence in Mainland China. We also obtained the cluster map based on the *RR* risk map to show which regions have significant clusters of high-risk hot spot and low-risk cold spot, as shown in Figure 3b. Supplemental File 4 includes the detailed method of the spatial cluster analysis (Local Moran’s I).

The *RR* map in Figure 3a shows prominent spatial aggregation characteristics, which suggested that spatial autocorrelation was useful when applied to disease incidence in modeling. For relative risk of HFMD in the whole Mainland of China, we identified six high-risk hot spots (high–high cluster) in which officials need to pay more attention in practice, as well as several low-risk cold spots (low–low cluster) shown in Figure 3b. Specifically, we found that very high-risk regions were concentrated in the southern part of North China (Beijing, Tianjin, and Hebei), South China (Guangdong and Guangxi), coastal areas of East China (Jiangsu and Shanghai), Southwest China (Sichuan and Chongqing junctions), Northwest China (Qinghai, Gansu, and Ningxia junctions), and Northeast China. In addition, high–low and low–high regions were outliers, but there were only a few in Figure 3b, which were also distributed very heterogeneously.

### 3.5. Estimated Spatiotemporal SMR Maps

**Figure 4:**
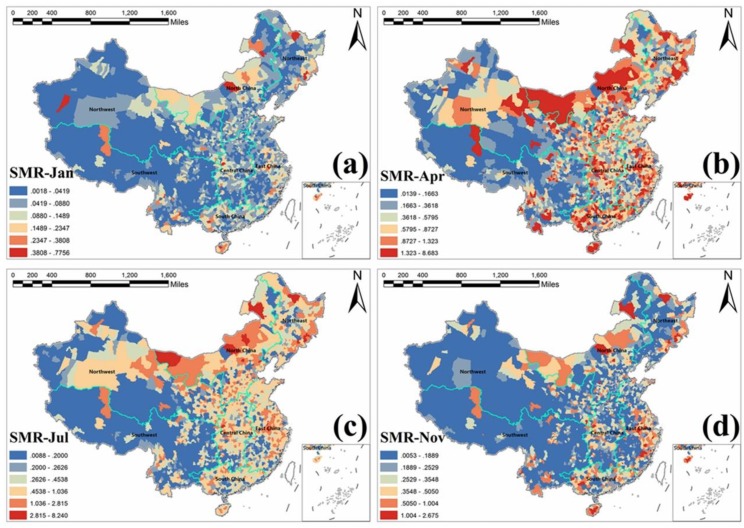
Estimated standard morbidity ratio (SMR) maps of HFMD incidence at county-level in Mainland China in 2009; (**a**) January, (**b**) April, (**c**) July, and (**d**) November.

Finally, we obtained the estimated county-level standard morbidity ratio (SMR) maps of HFMD incidence in Mainland China across 12 months of the year 2009. Figure 4 illustrates the estimated spatial SMR maps for four months. SMR was also explained by relative risk, characterized by values around 1. Compared with the original HFMD children cases maps (Figure 1) with a lot of zero-value areas, the SMR map of Figure 4a not only maintained the original spatial risk distribution, but also captured the local risk variation of those zero-value areas. In addition, the risk distributions of HFMD incidence were different among those four months in Figure 4. April (Figure 4b) had the highest risk, followed by July (Figure 4c), while January (Figure 4a) and November (Figure 4d) had relatively lower risks. SMR maps could give people hide (zero-value region) and more intuitive (remove and smooth the extreme outliers) information for disease prevention and control. The applied spatiotemporal ZINB model was approved effective to solve the ZI problem and generate complete spatiotemporal SMR maps.

## 4. Discussion

China’s HFMD epidemic data suffer from a serious zero-inflated problem, but to our best knowledge, most of the previous HFMD studies [16,17,23,24,25] ignored it, which could bring unknown errors and uncertainties for environmental epidemiology analysis and disease mapping [26,32,33]. Our study is the first one to consider the zero-inflated effect in spatiotemporal modeling for a comprehensive spatiotemporal risk assessment and mapping relative risk for HFMD incidence in the entire Mainland China at a fine-scale county level.

First of all, a main contribution of our study is that, under the spatiotemporal assessment framework, we gave evidence to confirm both climate and socioeconomic factors had significant influences on HFMD incidence across China.

Regarding the climate aspect, our results were consistent with the previous studies [4,5,6,7], that a hot and humid climate was an ideal environment for HFMD. Prior work has only confirmed that climate variables are risk factors for HFMD at a spatial [16,17] or temporal scale [10,19], but not at the spatiotemporal scales. Moreover, we found that increased sunshine hours and wind speed were also positively related to HFMD occurrence. Possible explanations are that more sunshine hours increase the surface temperature, encouraging people to spend more time outdoors, which can facilitate contact for disease transmission, and higher wind speed accelerates the spread of the virus.

Regarding the socioeconomic aspect, our study further confirmed that economic development was positively correlated with the occurrence of HFMD. This finding is consistent with previous studies of HFMD using different socioeconomic variables, such as GDP [17,55], children of rural-to-urban migrant workers [56], and urban areas in comparison with rural areas [57]. In urban areas, the higher population density leads to easy spreading of the virus [55]. Most children in the developed regions of China go to daycares or kindergartens, whereas children in undeveloped areas usually stay at home where there is less of a chance of being in contact with HFMD-infected children [18]. Our finding, that higher proportion of children also had a higher risk, is consistent with other studies [16,17,58], which indicated that children population agglomeration could increase disease risk.

Secondarily, another important contribution of this study was that we detected new characteristics of spatial and temporal risk variations for HFMD incidence on the local scale.

In the temporal dimension, the HFMD outbreak in Mainland China has obvious seasonal characteristics. Throughout the year, our results indicated that the highest risk occurred at the beginning of summer, which is consistent with other studies [2,59]. One possible reason is that hot and humid environments in summer make it easier for the virus to survive and spread. More importantly, we further found that there was an increasing risk trend from fall to winter, which is seldom identified [11] for HFMD. This indicated that cold and dry environments may also be risky for HFMD spread.

In the spatial dimension, the *RR* and hotspot mapping results showed important implications of strong spatial clustered patterns of HFMD risk assessment in Mainland China. We also found that a relative risk of HFMD incidence in the eastern part of China was more obvious than in the western and even some central parts of China. It may be because of the fact that Eastern China is located in the East Asian monsoon region, with the highest precipitation along the coastal region gradually declining inward [60]. As a result, Eastern China is more humid, a key risk factor for HFMD, compared with Western and Central China. Moreover, the population in Eastern China is much denser than that in Western and Central China, increasing the chance of HFMD infections. In addition, there was strong spatial heterogeneity other than spatial autocorrelation in some low-risk regions, such as the central part of China, while the actual risks were relatively high.

Moreover, this study demonstrated the advantages of the applied spatiotemporal zero-inflated model. We found that spatiotemporal ZI models had better performance than traditional spatiotemporal models, which indicates that it is necessary to account for zero-inflated effects in modeling, especially for disease data with serous ZI problems. We also found that negative binomial data prior is better than Poisson data prior for both spatiotemporal ZI models and traditional spatiotemporal models in our case. This may be because of the presence of overdispersion in China’s HFMD data. As our study focused on the smallest county-level units, it would lead to strong differences across all of China (as shown in Figure 1), which is a possible cause of overdispersion. For disease data with the ZI problem and overdispersion distribution, we suggest using the ZINB model to replace the traditional epidemic Poisson model, in order to improve model fitness and prediction.

Eventually, regarding disease mapping, this is the first study to generate the complete spatiotemporal SMR risk maps of HFMD at a fine scale (i.e., county-level) in the whole Mainland China, accounting for the ZI influence. With these local SMR maps, we could further analyze the risk differences in each spatial county and temporal frame, even in those zero-inflated regions, which is of great significance for the prevention and control of local disease transmission. 

The limitations in this study are as follows. First, the socioeconomic data used in this study do not contain any temporal changes, as the data are the summation of one year. Second, there might be unreported HFMD cases, because of the individual disease severity and the gaps between levels of regional medical resources [58], but we were not able to obtain tangible information about underreporting [16]. Moreover, the applied ZI model cannot examine how or which covariates significantly affect the non-occurrence ZI regions [61,62], which should be further studied. At last, this study did not consider environmental variables, such as soil, land cover, and air pollution [63,64], which could potentially influence HFMD. Future work with more environmental variables may offer new insights into HFMD risk assessment.

## 5. Conclusions

In this study, we applied the advanced spatiotemporal ZINB model under the BHM framework to first account for zero-inflated influence for HFMD spatiotemporal epidemic analysis and disease mapping. We found the spatiotemporal ZINB model was better fitted for China’s HFMD cases than other comparative models. We confirmed that under spatiotemporal scales, both climate and socioeconomic variables had significant influences on the HFMD incidence. Our findings also revealed the temporal nonlinear (seasonal) and spatial autocorrelation (hot spots) features of HFMD in China. The first complete spatiotemporal risk maps of HFMD generated by this study provides a better understanding of influencing factors, distribution, and transmission for HFMD in China at the local scale. Our applied spatiotemporal ZINB model could be an efficient way to solve the zero-inflated problem for spatiotemporal assessment in environmental health and epidemiology and applied to other regions for risk assessment of infectious diseases and disease mapping.

## Figures and Tables

**Table 1:** Evaluation results of the alternative spatiotemporal Bayesian models for the hand, foot, and mouth disease (HFMD) case of China.

Model	*DIC*	*P_D_*	*LS*	*WAIC*	*P_W_*
model 1	352883	2112	7.19	381936	27738
model 2	317909	1983	6.55	343243	24113
model 3	152998	1948	2.94	153404	2022
model 4	151201	1934	2.87	151543	1982

Notes: (model 1: Poisson; model 2: zero-inflated Poisson (ZIP); model 3: Negative binomial; model 4: zero-inflated negative binomial (ZINB)). *DIC*: deviance information criterion; *P_D_*: effective number of parameters for *DIC*; *LS*: logarithmic score; *WAIC*: Watanabe-Akaike information criterion; *P_w_*: effective number of parameters for *WAIC*.

**Table 2:** Estimated posterior parameters and relative risk (*RR*) values of the climate and socioeconomic risk factors on HFMD incidence.

Variables Name	Mean	0.025 CI	0.975 CI	SD	*RR*
Temperature	0.7053	0.6664	0.7441	0.0198	2.02
Relative humidity	0.1112	0.0682	0.1542	0.0219	1.12
Wind speed	0.2150	0.1791	0.2510	0.0183	1.24
Sunshine hours	0.1444	0.1017	0.1871	0.0218	1.16
Proportion of children	0.1344	0.0208	0.2480	0.0579	1.14
Enterprise number density	0.3406	0.2180	0.4631	0.0624	1.41
Per capita gross domestic product (GDP)	0.1970	0.0841	0.3098	0.0575	1.22
Per capita fixed assets investment	0.3637	0.2517	0.4755	0.0570	1.44

## Supplementary Materials

The following are available online at http://www.mdpi.com/1660-4601/15/7/1476/s1, File 1: Climate and socioeconomic variables (Table S1: Index of climate and socioeconomic variables.), File 2: Modeling R codes, File 3: Covariates selection results (Table S2: Results of multicollinearity evaluation, Table S3: Results of the forward stepwise regression, Table S4: *DIC* evaluation.), File 4: Spatial cluster mapping (Moran’s I analysis).
