# Supplementary material for: Risk Assessment and Mapping of Hand, Foot, and Mouth Disease at the County Level in Mainland China Using Spatiotemporal Zero-Inflated Bayesian Hierarchical Models
Source: Int J Environ Res Public Health. 2018 Jul 12;15(7):1476. doi: 10.3390/ijerph15071476 (PMC6069258; doi:10.3390/ijerph15071476)
Supplement: Supplementary file 1 [file ijerph-15-01476-s001.pdf]

SUPPLEMENTARY MATERIAL

Supporting Information for the following paper

Title:

Risk Assessment and Mapping of Hand, Foot, and Mouth Disease at the County Level in Mainland China Using Spatiotemporal Zero-Inflated Bayesian Hierarchical Models

Authors:

Chao Song, Yaqian He, Yanchen Bo*, Jinfeng Wang, Zhoupeng Ren, Huibin Yang

* **Corresponding author**: Y.B. (boyc@bnu.edu.cn).

Content

1. Climate and socioeconomic variables.. 2

2. Modeling R codes 3

3. Covariates selection results.. 4

4. Spatial cluster mapping (Moran's I analysis) 2

1. Climate and socioeconomic variables

We collected 20 potential variables accounting for both climate and socioeconomic aspects in this study as potential environmental factors for HFMD in Mainland China, see Table S1. The monthly climate data were provided by the China Meteorological Data Sharing Service System [1]. The socioeconomic factors were from the China County Statistical Yearbook, China Statistical Yearbook for Regional Economy, and China City Statistical Yearbook of 2009 [2]. All the variables were standardized to be dimensionless using z-score standardization in R software.

Table S1. Index of climate and socioeconomic variables.

Climate Variables	X	Socioeconomic Variables	SE
Temperature	X1	Per capita hospital bed number	SE1
Relative humidity	X2	Child population density	SE2
Precipitation	X3	Proportion of children	SE3
Air pressure	X4	Children gender ratio	SE4
Wind speed	X5	Population density	SE5
Sunshine hours	X6	Enterprise number density	SE6
		Per capita industrial output values	SE7
		Per capita number of telephone calls	SE8
		Per capita household savings	SE9
		Average wage of workers	SE10
		Population density of primary school students	SE11
		Per capita gross domestic product (GDP)	SE12
		Per capita consumption	SE13
		Per capita fixed assets investment	SE14

2. Modeling R codes

The core codes in R for modeling the applied spatiotemporal models (model 1: Poisson; model 2: ZIP; model 3: Negative binomial; model 4: ZINB) in this study are summarized as below. For more coding details, please see [3-5].

```
library(INLA)

# model 1
formula.model1<- y~ 1 + X1+X2+X5+X6 + SE3+SE6+ SE12+SE14 +
f(ID.area,model="bym",graph=studyAreaMap.adj) +
f(ID.month,model="rw1")+ f(ID.month1,model="iid")
Results.model1<-inla(formula.model1,family="poisson",data=data,E=E,
control.compute=list(dic=TURE,cpo=TRUE,waic=TURE),
control.inla = list(strategy="simplified.laplace"))

# model 2
formula.model2<- y~ 1 + X1+X2+X5+X6 + SE3+SE6+ SE12+SE14 +
f(ID.area,model="bym",graph=studyAreaMap.adj) +
```

```
f(ID.month,model="rw1")+ f(ID.month1,model="iid")
Results.model2<-inla(formula.model2,family="zeroinflatedpoisson1",data=data,E=E,
control.compute=list(dic=TRUE,cpo=TRUE,waic=TURE),
control.inla = list(strategy="simplified.laplace"))

# model 3
formula.model3<- y~ 1 + X1+X2+X5+X6 + SE3+SE6+ SE12+SE14 +
f(ID.area,model="bym",graph=studyAreaMap.adj) +
f(ID.month,model="rw1")+ f(ID.month1,model="iid")
Results.model2<-inla(formula.model3,family="nbinomial ",data=data,E=E,
control.compute=list(dic=TRUE,cpo=TRUE,waic=TURE),
control.inla = list(strategy="simplified.laplace"))

# model 4
formula.model4<- y~ 1 + X1+X2+X5+X6 + SE3+SE6+ SE12+SE14 +
f(ID.area,model="bym",graph=studyAreaMap.adj) +
f(ID.month,model="rw1")+ f(ID.month1,model="iid")
Results.model4<-inla(formula.model4,family="zeroinflatednbinomial2",data=data,E=E,
control.compute=list(dic=TRUE,cpo=TRUE,waic=TURE),
control.inla = list(strategy="simplified.laplace"))
```

3. Covariates selection results

Covariates selection results of the climate and socioeconomic variables accounting for multicollinearity [6], significance [7], and *DIC* [8] are summarized in this section.

First, we removed four variables (SE 2, 5, 6, and 11) with higher VIF according to the screening criteria VIF < 10, see Table S1.

Table S2. Results of multicollinearity evaluation.

Variables	VIF	Selection	Variables	VIF	Selection
SE10	1.281	Y	X3	3.693	Y
SE4	1.555	Y	X6	3.793	Y
X5	1.819	Y	SE12	4.087	Y
X2	1.857	Y	SE6	4.243	Y
X4	1.92	Y	SE13	4.366	Y
SE1	2.155	Y	SE7	5.482	Y
SE14	2.346	Y	SE9	10.931	N
X1	2.437	Y	SE11	16.557	N
SE3	2.451	Y	SE5	62.073	N
SE8	2.495	Y	SE2	75.65	N

Second, we built the forward stepwise regression models to maintain 10 variables with statistical significance (sig. < 0.05), see Tables S3. B is the regression coefficient, T is the t-test values and sig. is the significance.

Table S3. Results of the forward stepwise regression

Covariate	B	T	Sig.
X1	0.173	21.860	0.000
SE3	−0.137	−19.426	0.000
X6	0.098	13.120	0.000
SE14	0.066	7.958	0.000
SE10	−0.055	−8.787	0.000
X5	0.037	4.973	0.000
X2	0.040	5.302	0.000
SE13	0.041	3.796	0.000
SE12	−0.039	−3.808	0.000
SE6	0.026	2.633	0.008

At last, we removed two variables (SE 10 and 13) with DIC change less than 30 units, see Table S4. The more the DIC value decreases, the more important the covariate is.

Table S4. *DIC* evaluation.

Removed Covariate Each Time	*DIC*	*DIC* Decrease	Selection
X1	641128.3	10172.0	Y
SE3	638050.3	7094.0	Y
X6	634126.4	3170.1	Y
SE14	633750.0	2793.7	Y
X5	632973.0	2016.7	Y
X2	632117.8	1161.6	Y
SE6	632079.0	1122.7	Y
SE12	631792.5	836.3	Y
SE10	630977.5	21.3	N
SE13	630957.9	1.6	N
Reference(all covariates)	630956.2	0.0	/

As a result, a total of eight variables including four climate (i.e., X 1, 2, 5 and 6) and four socioeconomic factors (i.e., SE 3, 6, 12 and 14) were selected as covariates for modeling.

4. Spatial cluster mapping (Moran's I analysis)

We applied the "Cluster and Outlier Analysis" in ArcGIS 10.2 software [9] to calculate the "Local Moran's I" statistic for spatial RR map in this study, in order to get clustered maps for further analysis. The local Moran's I statistics is given as [10]:

$$I_i = \frac{x_i - \overline{X}}{S_i^2} \sum_{j=1, j \neq i}^{n} w_{i,j}(x_j - \overline{X}) \tag{1}$$

where x_{i} is the RR value for spatial unit *i*, $w_{i,j}$ is the spatial weight between spatial unit *i* and *j*, n

is total number of all spatial units in the map. $\overline{X}$ and S_i^2 are calculated as:

$$\overline{X} = \frac{\sum_{j=1}^{n} x_j}{n} \quad (2)$$

$$S_i^2 = \frac{\sum_{j=1, j\neq i}^{n} (x_j - \overline{X})^2}{n-1} \quad (3)$$

A positive value for *I* indicates that a feature has neighboring features with similarly high or low attribute values; this feature is part of a cluster. A negative value for I indicates that a feature has neighboring features with dissimilar values; this feature is an outlier. Based on this interpolation, the tool distinguishes between a statistically significant cluster of high values (HH), cluster of low values (LL), outlier in which a high value is surrounded primarily by low values (HL), and outlier in which a low value is surrounded primarily by high values (LH).

Reference

1. Bo, Y.; Song, C.; Wang, J.; Li, X. Using an autologistic regression model to identify spatial risk factors and spatial risk patterns of hand, foot and mouth disease (HFMD) in mainland China. *BMC Public Health* **2014**, *14*, 358.
2. Song, C.; Yang, X.; Shi, X.; Bo, Y.; Wang, J. Estimating missing values in China's official socioeconomic statistics using progressive spatiotemporal Bayesian hierarchical modeling. *Sci. Rep.* **2018**, *8*, 10055.
3. Ugarte, M.D.; Adin, A.; Goicoa, T.; Militino, A.F. On fitting spatio-temporal disease mapping models using approximate Bayesian inference. *Stat. Methods Med. Res.* **2014**, *23*, 507–530.
4. Blangiardo, M.; Cameletti, M.; Baio, G.; Rue, H. Spatial and spatio-temporal models with R-INLA. *Spat. Spatio-Temporal Epidemiol.* **2013**, *7*, 39–55.
5. Schrödle, B.; Held, L. Spatio-temporal disease mapping using INLA. *Environmetrics* **2011**, *22*, 725–734.
6. Vatcheva, K.P.; Lee, M.; McCormick, J.B.; Rahbar, M.H. Multicollinearity in regression analyses conducted in epidemiologic studies. *Epidemiology (Sunnyvale, Calif.)* **2016**, *6*, 227.
7. Cronk, B.C. How to use spss®: A step-by-step guide to analysis and interpretation. *Routledge*: Abingdon, UK, 2017.
8. Burnham, K.P.; Anderson, D.R. Multimodel inference: Understanding AIC and BIC in model selection. *Sociological Methods Res.* **2004**, *33*, 261–304.
9. Scott, L.M.; Janikas, M.V. Spatial statistics in ArcGIS. In Handbook of applied spatial analysis; *Springer*: New York, NY, USA, 2010; pp. 27–41.
10. Anselin, L. Local indicators of spatial association—LISA. *Geog. Anal.* **1995**, *27*, 93–115.
